# Exploring the Molecular Mechanism of Hydroxychloroquine Against IgAN Through Network Pharmacology, MD Simulations and Experimental Assessment

**DOI:** 10.1111/jcmm.70615

**Published:** 2025-05-26

**Authors:** Yuyuan Liu, Jinfang Hu, Jialing Wang, Yanzhe Wang, Gang Wu

**Affiliations:** ^1^ Department of Nephrology The Affiliated Suzhou Hospital of Nanjing Medical University, Suzhou Municipal Hospital, Gusu School, Nanjing Medical University Suzhou Jiangsu China; ^2^ Department of Nephrology Tongren Hospital, Shanghai Jiao Tong University School of Medicine Shanghai China; ^3^ Shanghai Tongren Hospital Bengbu Medical University Bengbu China

**Keywords:** hydroxychloroquine, IgA nephropathy, molecular dynamics simulation, network pharmacology, PTGS2

## Abstract

Hydroxychloroquine (HCQ) has recently been reported to be an effective treatment for IgA nephropathy (IgAN); however, the exact mechanism remains elusive. This study aimed to explore the molecular mechanisms of HCQ against IgAN. IgAN‐related genes and HCQ target sets were screened from online databases, and a section of them was identified as targets of HCQ against IgAN. In total, 1575 IgAN‐ related genes, 415 HCQ targets, and 125 targets of HCQ against IgAN were identified. The results of the enrichment analysis showed that the targets of HCQ against IgAN were related to inflammation and immune response related pathways. The PPI network and subnetworks identified prostaglandin‐endoperoxide synthase 2 (PTGS2) as the main seed gene. Molecular docking and molecular dynamic (MD) simulations revealed that HCQ could well bind to the PTGS2 protein. Furthermore, clinical data indicated that PTGS2 was overexpressed in patients with IgAN and was negatively correlated with estimated glomerular filtration rate (eGFR). Moreover, consistent with the effect of meloxicam, a PTGS2 inhibitor, HCQ could decrease the expression of PTGS2 and profibrotic proteins in the IgAN cell model. Consequently, HCQ can mediate inflammation and immune response regulation via multiple pathways and targets, among which PTGS2 is probably the key target of HCQ against IgAN.

## 1 Introduction

IgA nephropathy (IgAN) was first described more than 50 years ago and has since become the most prevalent primary glomerulonephritis worldwide [1]. Up to 30%–40% of patients with IgAN progress to end‐stage kidney disease within 20 years of diagnosis, making IgAN one of the most common causes of kidney failure globally [2]. However, the precise pathogenesis of IgAN remains elusive, and there are no targeted treatment strategies [3]. In most cases, therapies for IgAN are still supportive, including blood pressure control and proteinuria remission. For IgAN patients with persistently significant proteinuria and a high risk of renal function decline, immunosuppressive drugs such as corticosteroids can be considered; however, these drugs have significant side effects [4].

Recently, many studies revealed that hydroxychloroquine (HCQ) could effectively reduce proteinuria in IgAN patients and delay the progression of IgAN without significant adverse events [5, 6, 7]. HCQ is a classical antimalarial drug used to treat autoimmune diseases, including systemic lupus erythematosus (SLE) and rheumatoid arthritis (RA) [8, 9]. It is generally considered that HCQ exerts anti‐inflammatory and immune regulatory roles by interfering with lysosomal activity and autophagy, interacting with membrane stability, and altering signalling pathways and transcriptional activity [9]. Limited research data have shown that HCQ treatment can suppress nuclear factor kappa B (NF‐κB) signalling and nod‐like receptor pyrin 3 (NLRP3) inflammasome activation and regulate the differentiation of CD4+ T cell subsets in the kidneys of IgAN rats, thereby mediating anti‐inflammatory and immune‐regulatory functions [10, 11]. Recent studies have also revealed that HCQ can inhibit macrophage activation and the PI3K/Akt signalling pathway, thereby alleviating renal fibrosis in different models [12, 13]. Meanwhile, renal fibrosis is one of the most important elements contributing to the progression of IgAN [14]. However, the exact mechanism by which HCQ delays the progression of IgAN remains unknown.

**FIGURE 1:**
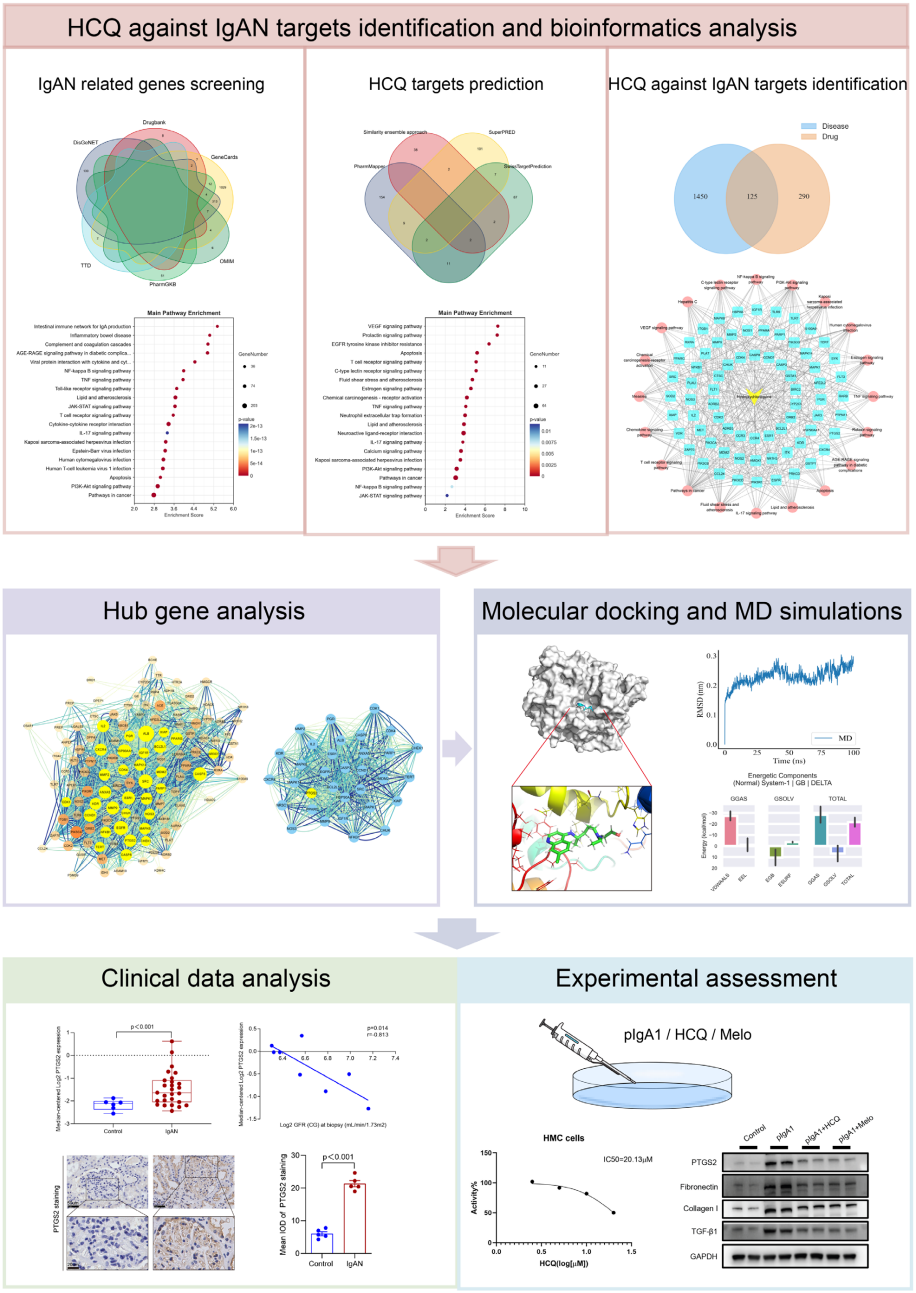
The graphical abstract of present study. This study first screened HCQ against IgAN targets with online database, among which hub genes were identified by PPI network. Then, molecular docking and MD simulations were performed to verify the reliability of HCQ binding to the protein of hub genes. Additionally, clinical data analysis and cell experiments indicated that PTGS2 is probably the key target of HCQ against IgAN.

Network pharmacology is a novel method that systematically studies the potential targets and pharmacological effects of a medicine via multiple gene, target, and pathway analyses [15]. Molecular docking and molecular dynamic (MD) simulations are commonly used techniques in computer‐aided drug research. They theoretically simulate the interactions between receptors and drug molecules to predict the binding mode and affinity of the drugs [16, 17]. In this study, we first used network pharmacology, molecular docking, and MD simulations technologies to preliminarily explore the potential targets and pathways of HCQ in the treatment of IgAN. Thereafter, we used clinical data to verify the expression of PTGS2 as a key target in patients with IgAN and performed in vitro experiments to further investigate the potential mechanism of HCQ against IgAN. This provides a scientific basis for the clinical application of HCQ in IgAN. The schematic diagram of our present study was presented in Figure 1.

## 2 Material and Methods

### 2.1 Ethics Statement

This study was approved by the Ethics Committee of the Affiliated Suzhou Hospital of Nanjing Medical University, Suzhou Municipal Hospital (Approval No. 2022‐117). All studies related to humans received written consent from the participants.

### 2.2 Human Renal Tissue Collection

All patients were recruited from the Affiliated Suzhou Hospital of Nanjing Medical University, Suzhou Municipal Hospital, China. The renal cortex of the IgAN patient group was obtained from patients who underwent renal biopsy and were diagnosed with IgAN, whereas the non‐diseased renal cortex from renal carcinoma patients who underwent nephrectomy was used as controls. After renal biopsy or surgery, renal tissues were collected for subsequent studies.

### 2.3 Identification of IgAN Related Genes

The IgAN‐related genes were screened using six databases, including Genecards (https://www.genecards.org/), Online Mendelian Inheritance in Man (OMIM, https://omim.org/), PharmGkb (https://www.pharmgkb.org/), Therapeutic Target Database (TTD, http://db.idrblab.net/ttd/), DisGeNet (https://www.disgenet.org/home/) and DrugBank (https://www.drugbank.ca/). Briefly, we used ‘IgA Nephropathy’ as the search term to search in the above databases. Then we established the IgAN‐related gene set by combining all screening results.

### 2.4 Prediction of HCQ Targets

The PubChem database (https://pubchem.ncbi.nlm.nih.gov/) was used to obtain SMILES information and spatial conformation for HCQ. Then, the SMILES information was imported into the SwissTargetPrediction (http://www.swisstargetprediction.ch/), Super‐PRED (https://prediction.charite.de/index.php) and similarity ensemble approach (SEA, https://sea.bkslab.org/) databases, and the spatial conformation was imported into PharmMapper (http://www.lilab‐ecust.cn/pharmmapper/) database for target prediction. As for SwissTargetPrediction, the target of HCQ was screened by limiting the species to ‘ *Homo sapiens* ’, and those with the probability > 0 was chosen. Finally, we combined the targets obtained from the four databases as the HCQ target set.

### 2.5 Go Function and Pathway Enrichment Analysis

Gene Ontology (GO) classification and Kyoto Encyclopedia of Genes and Genomes (KEGG) pathway enrichment analyses were performed using the clusterProfiler package as previously described [18]. Statistical significance was set at *p* < 0.01.

### 2.6 Drug‐Targets‐Pathway Network Construction

Targets of HCQ against IgAN were obtained by intersecting the IgAN‐related gene set and the HCQ target gene set. Bioinformatics analysis of target genes was performed using Metascape [19]. The drug‐targets‐pathways (top 20) network was constructed and visualised using the Cytoscape software (version 3.8.0).

### 2.7 Protein–Protein Interaction (PPI) Network Construction

To construct the PPI network, the target genes were imported into the STRING database (version 11.0, https://string‐db.org/). By defining organisms as ‘ *Homo sapiens* ’ and confidence as 0.4 (medium confidence), the resulting PPI network was established. Subsequently, the Cytoscape software (version 3.8.0) was used to visualise the PPI network.

### 2.8 Cluster Analysis of the PPI Network and Seed Gene Identification

The MCODE plug‐in in Cytoscape was used to screen PPI network modules and seed genes using various cut‐offs: degree = 2, k‐core = 2, node score = 0.2, and maximum depth = 100 [20].

### 2.9 Molecular Docking and MD Simulations

To explore the interaction between HCQ and proteins of hub gene, molecular docking was performed using AutoDock software. Moreover, to investigate the dynamic interactions between HCQ and the PTGS2 protein, MD simulations were conducted using Gromacs2019.6 software combined with the amber14sb force field. A cubic water box consisting of a TIP3P water model was used to solvate the system and sodium ions were subsequently added. Before MD simulations, the energy of the entire system was optimised using the steepest descent method with 5000 steps. Additionally, to compute the van der Waals and Coulomb energies, we set the cutoff distance to 1.4 nm. During the MD simulations, the V‐rescale temperature coupling method was used to control the temperature to 300 K, and the Berendsen method was used to control the pressure to 1 bar. When the temperature reached 300 K, the coupling process was performed by the double equilibration method using a conserved number of particles (N), system volume (V), temperature (T) (NVT), and a constant number of particles (N), system pressure (P), and temperature (T) (NPT) for 30 ps each. Finally, a 100 ns MD simulation was performed. Based on the MD simulation results, the root mean square deviation (RMSD), radius of gyration (RG), and solvent accessible surface area (SASA) were calculated and analysed.

### 2.10 Binding Free Energies and Energy Components Computation

To compute the binding affinity of HCQ for PTGS2, the GROMACS tool, which is based on the gmx_MMPBSA method [21], was applied. The binding free energies for the complexes were calculated according to the following equations:

$${\mathrm{\Delta G}}_{\text{bind}}={\mathrm{\Delta E}}_{\text{complex}}–\left({\mathrm{\Delta G}}_{\text{receptor}}+{\mathrm{\Delta G}}_{\text{ligand}}\right)$$

$$={\mathrm{\Delta E}}_{\mathrm{MM}}+{\mathrm{\Delta G}}_{\text{solv}}–\mathrm{T\Delta S}$$

$${\mathrm{\Delta E}}_{\mathrm{MM}}={\mathrm{\Delta E}}_{\text{internal}}+{\mathrm{\Delta E}}_{\text{elec}}+{\mathrm{\Delta E}}_{\mathrm{vdw}}$$

$${\mathrm{\Delta G}}_{\text{solv}}={\mathrm{\Delta G}}_{\mathrm{GB}}+{\mathrm{\Delta G}}_{\mathrm{SA}}$$

here, ${\mathrm{\Delta E}}_{\text{internal}}$ contains the bond energy (ΔE_bond_), angular energy (ΔE_angle_), and torsional energy (ΔE_torsion_), and is generally regarded as zero. TΔS was ignored in this study because of its high consumption of computing resources and low accuracy. ΔE_elec_, ΔE_vdw_, ΔG_GB_ and ΔG_SA_ represent the electrostatic, van der Waals, solvation free, and non‐polar solvation free energies, respectively.

### 2.11 Nephroseq

Data from the Nephroseq (www.nephroseq.org, University of Michigan; Ann Arbor, MI, USA) database were extracted to further examine gene expression in the human kidney. PTGS2 expression data were obtained from the datasets ‘Reich IgAN Glom’; and data of correlation analysis from the datasets ‘Cox IgAN Blood datasets.

### 2.12 Immunofluorescent and Immunohistochemical Staining

Renal biopsy surplus tissues from five patients diagnosed with IgAN via renal biopsy and renal tissues adjacent to cancer from 5 controls were subjected to immunofluorescence and immunohistochemistry (IHC) staining. IHC staining for PTGS2 (ab283574, 1:100; Abcam) and immunofluorescence staining for IgA (ab223410, 1:100; Abcam) were performed as previously described [22, 23]. Images were acquired using a microscope (Leica Microsystems, Mannheim, Germany). For the immunofluorescence assay results, qualitative analysis of IgA deposition in the glomerular mesangial area was performed, and representative images were selected for presentation. For the IHC staining results, quantitative analysis was performed using Image‐Pro Plus 6.0. Specifically, for each section, five random fields of view primarily containing glomeruli were selected, and the average mean integrated optical density (IOD) of these fields was calculated to represent the mean IOD value for that sample.

### 2.13 Cell Culture and Treatment

The human mesangial cell line (HMC) was purchased from the FuHeng Biology Co. (Shanghai, China). Cells were cultured in DMEM medium (Gibco, USA), supplemented with 10% fetal bovine serum (FBS; Gibco, USA), and incubated at 37°C in a humidified incubator with 5% CO_2_. Monomeric human IgA1(ab91020; Abcam) was heated and aggregated at 65°C for 150 min to gain polymer IgA1 (pIgA1) as previously described [24]. HCQ (Yuanye, China) and meloxicam (Sigma‐Aldrich, USA) were dissolved in dimethyl sulfoxide (DMSO) separately. For the in vitro studies, when the cells reached 50%–60% confluence, they were starved overnight and then treated with HCQ (5 μM) or meloxicam (5 μM) [25] and the indicated concentration of pIgA1 for 48 h. Subsequently, the cells were harvested for further study, such as western blotting.

### 2.14 Cell Proliferation Assay

In vitro, pIgA1 can stimulate the proliferation of mesangial cells to establish a cellular model of IgA nephropathy [24]. To determine the optimal concentration of pIgA1 for stimulation, the proliferation of HMCs treated with different concentrations of pIgA1 was detected using a cell counting kit‐8 (CCK‐8) assay (Beyotime, China). Briefly, cells were inoculated at a density of 2 × 10^3^ cells/well in a 96‐well plate. After cell adherent growth, the medium was replaced with fresh medium containing various concentrations of pIgA1 (0, 12.5, 25, 50 ug/mL) and incubated for 48 h. Then, 10 μL of CCK‐8 solution was added to every well and incubated at 37°C for 1 h. Next, each well's optical density (OD) was detected at 450 nm with the spectrophotometer (Thermo Fisher Scientific, USA). The tested OD values were collected for statistical analysis.

### 2.15 Cell Viability Assay

To determine the cytotoxicity of HCQ, the viability of HMCs treated with different concentrations of HCQ was detected by CCK‐8 assay. Firstly, cells were inoculated at a density of 5 × 10^3^ cells/well in a 96‐well plate. After cell adherent growth, the medium was replaced with fresh medium containing various concentrations of HCQ (2.5, 5, 10, 20 μM) and incubated for 48 h. Subsequently, the CCK‐8 assay was performed, following the same procedures as the cell proliferation assay. Then, the measured OD values were calculated for cell viability according to the following formula: Cell viability (%) = (Experimental group OD value − Blank control group OD value)/(Control group OD value − Blank control group OD value) × 100%. Finally, the cell viability values were imported into GraphPad Prism 9.0 for half‐maximal inhibitory concentration (IC50) calculation.

### 2.16 Western Blotting

Western blotting was performed as previously [26]. Briefly, total proteins were extracted from cells with RIPA lysate (Beyotime, China), and protein concentrations were quantified by the bicinchoninic acid (BCA) protein assay kit (Beyotime, China). Protein samples were loaded onto a 4%–12% polyacrylamide gel (GenScript, USA), electrophoresis was performed, the samples were transferred to a polyvinylidene fluoride (PVDF) membrane, and the membrane was sealed with 5% skimmed milk. Then, the membranes were incubated overnight at 4°C with primary antibodies, including PTGS2 (ab283574, 1:1000; Abcam), fibronectin (ab2413, 1:1000; Abcam), collagen I (ab260043, 1:1000; Abcam), TGF‐β1 (ab215715, 1:1000; Abcam) and GAPDH (AF1186, 1:1000; Beyotime), followed by incubation with a secondary antibody (ab6721, 1:5000; Abcam) at room temperature for 1 h. The immunoblots were visualised with enhanced chemiluminescence. Finally, protein expression was quantified with the Image J software, and GAPDH was used as a reference.

### 2.17 Statistical Analysis

SPSS version 22.0, and GraphPad Prism 9 were used for data analysis and graph drawing. Data are expressed as mean ± SEM or median and interquartile range (IQR). The differences in expression were analysed using a two‐tailed unpaired Student's *t*‐test or one‐way ANOVA with LSD or Tamhane's T2 test. For Nephroseq data, statistical significance was computed using Welch's *t*‐test and *q*‐values (exported from Nephroseq). Differences were considered statistically significant at *p* < 0.05.

## 3 Results

### 3.1 Screening of IgAN Related Genes and Enrichment Analysis

**FIGURE 2:**
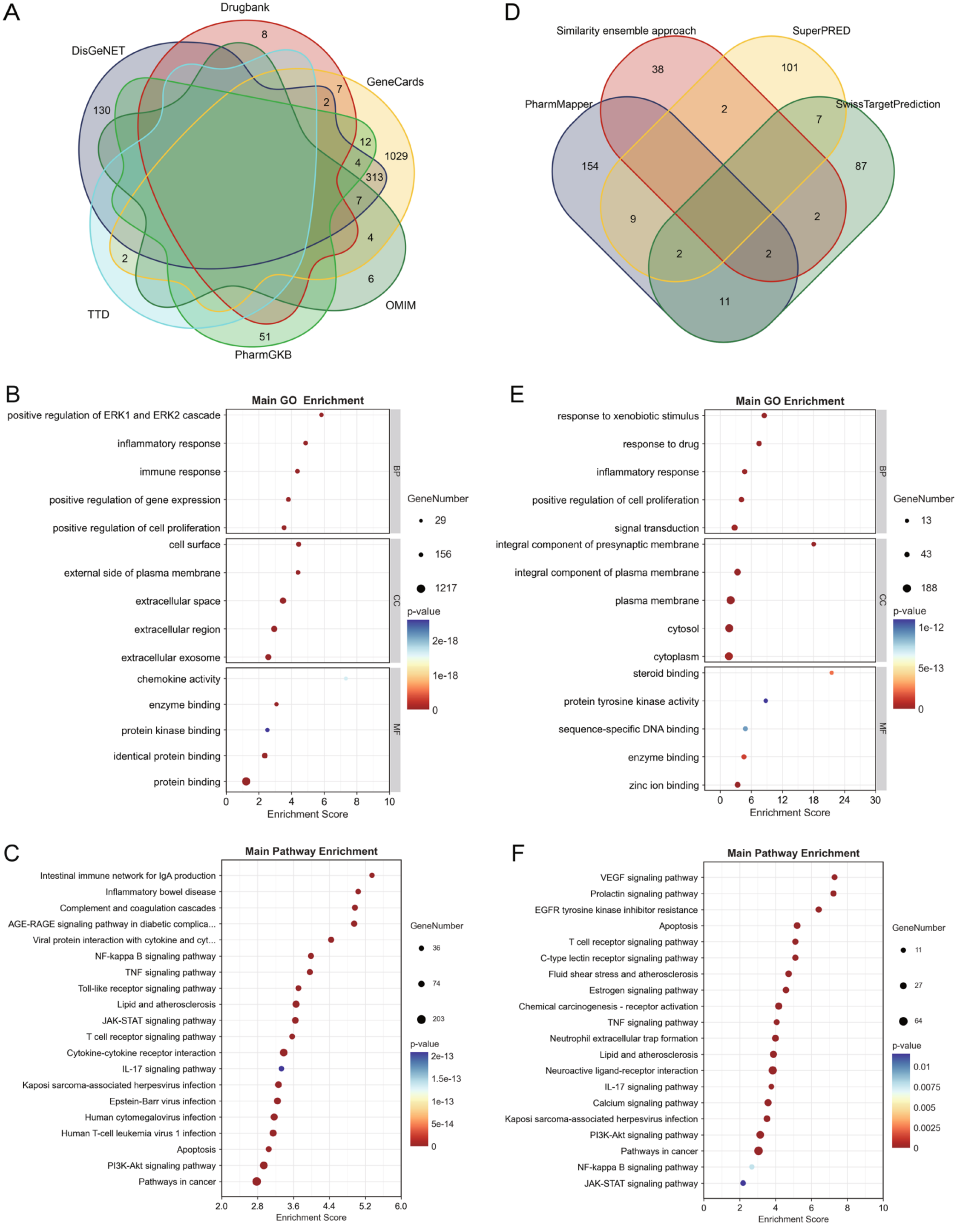
IgAN related genes and HCQ targeted proteins were screened and analysed. (A) Identification of the IgAN related genes by taking a union of all the results from 6 databases. (B, C) GO function and KEGG pathway analysis of IgAN related genes. (D) Pharmacological targets of HCQ were predicted by 4 databases. (E, F) GO function and KEGG pathway analysis of HCQ targets.

To investigate the pathogenesis of IgAN, disease‐related genes were screened using multiple databases. As presented in Figure 2A, 1575 IgAN related genes were identified. Furthermore, GO function analysis and KEGG pathway analysis were performed; under the conditions of *p* < 0.01, a total of 1554 GO terms and 152 KEGG pathways were obtained. For GO function analysis, the top 5 terms of biological process (BP), cellular component (CC), and molecular function (MF) were visualised in order of enrichment score, as shown in Figure 2B. Among them, BP mainly enriched in positive regulation of ERK1 and ERK2 cascade, inflammatory response, immune response, etc. CC mainly enriched in cell surface, external side of plasma membrane, extracellular space, etc. MF mainly enriched in chemokine activity, enzyme binding, protein kinase binding, etc. For KEGG pathway analysis, the main 20 pathways were visualised in order of enrichment score, as shown in Figure 2C. It showed that disease‐related genes were enriched in intestinal immune network for IgA production, NF‐kappa B signalling pathway, TNF signalling pathway, and PI3K‐Akt signalling pathway, which are closely related to inflammation and immune response [27, 28, 29].

### 3.2 Prediction of HCQ Targeted Proteins and Enrichment Analysis

To explore the mechanism of HCQ against disease, we predicted the targets of HCQ and performed further analyses. First, 415 pharmacological targets of HCQ were identified (Figure 2D). Then, GO function analysis and KEGG pathway analysis were performed; under the conditions of *p* < 0.01, a total of 553 GO terms and 129 KEGG pathways were obtained. For GO function analysis, the top 5 terms of BP, CC, and MF were visualised in order of enrichment score, as shown in Figure 2E. Among them, BP mainly enriched in response to xenobiotic stimulus, positive regulation of cell proliferation, inflammatory response, etc. CC mainly enriched in plasma membrane, integral component of plasma membrane, cytoplasm, etc. MF mainly enriched in steroid binding, protein tyrosine kinase activity, enzyme binding, etc. For KEGG pathway analysis, the main 20 pathways were visualised in order of enrichment score, as shown in Figure 2F. It showed that HCQ targets were enriched in the T cell receptor signalling pathway, C‐type lectin receptor signalling pathway, TNF signalling pathway, IL‐17 signalling pathway, and PI3K‐Akt signalling pathway, which play significant roles in inflammation and immune response regulation [29, 30, 31, 32].

### 3.3 Identification of the Potential Targets of HCQ Against IgAN and Bioinformatics Analysis

**FIGURE 3:**
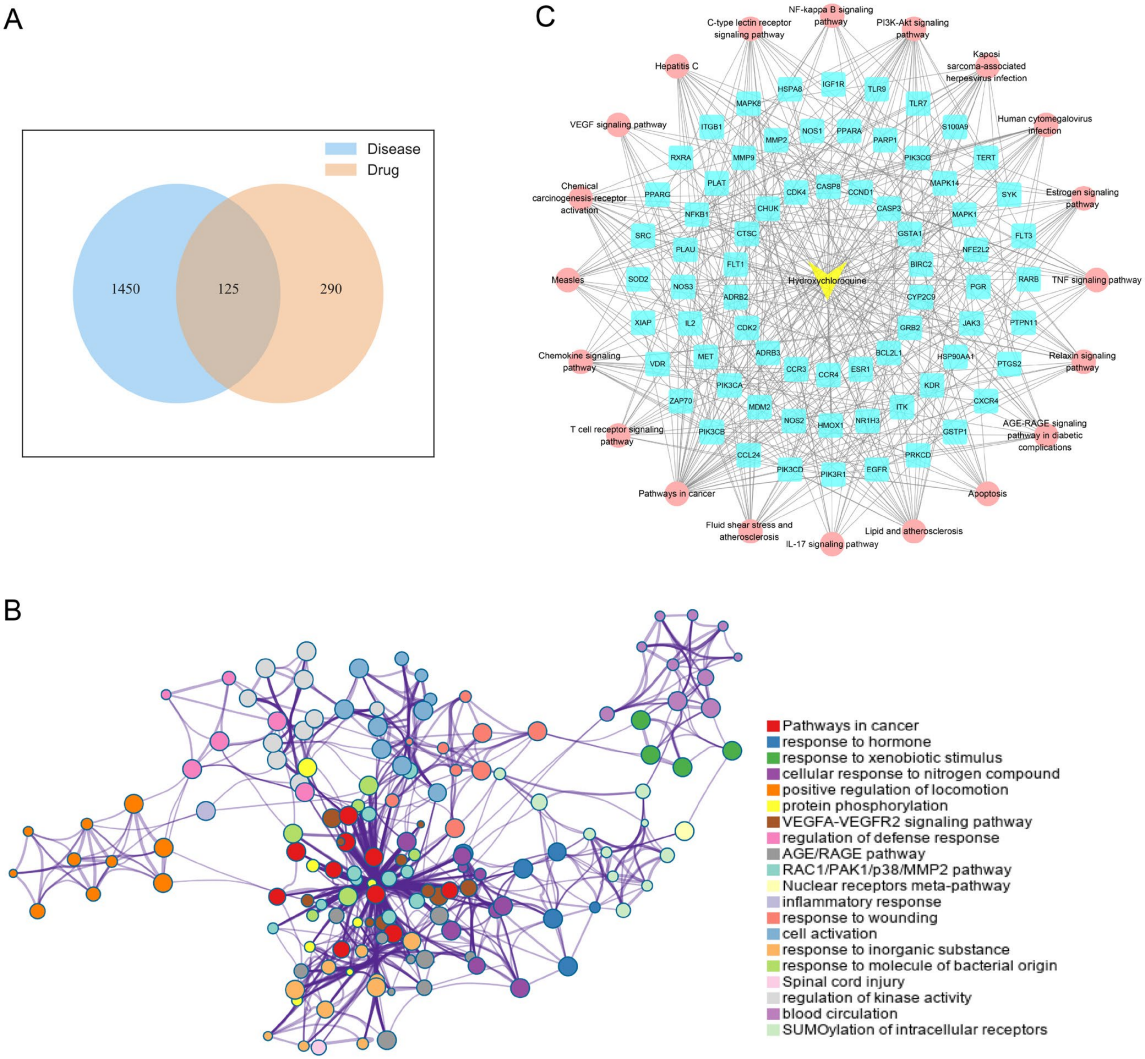
Identification of the potential targets of HCQ in the treatment of IgAN and bioinformatics analysis. (A) Venn diagram of disease related targets and drug targets. (B) Enrichment analysis of the intersecting target genes of disease and drug by Metascape online tool. (C) Drug‐targets‐pathways (top 20) network.

The results for HCQ and disease targets were intersected, and 125 targets of HCQ against IgAN were obtained (Figure 3A). Subsequently, enrichment analysis showed that these targets were mainly enriched in response to xenobiotic stimulus, the AGE/RAGE pathway, the RAC1/PAK1/p38/MMP2 pathway, inflammatory response, and regulation of kinase activity (Figure 3B). Furthermore, pathway analysis revealed that targets were mainly enriched in the C‐type lectin receptor signalling pathway, chemokine signalling pathway, PI3K‐Akt signalling pathway, TNF signalling pathway, and NF‐kappa B signalling pathway, which are closely related to inflammation and immune response regulation [28, 29, 32]. The top 20 drug‐target pathway networks were constructed and visualised in Figure 3C.

### 3.4 Screening of the Key Target of HCQ Against IgAN

**FIGURE 4:**
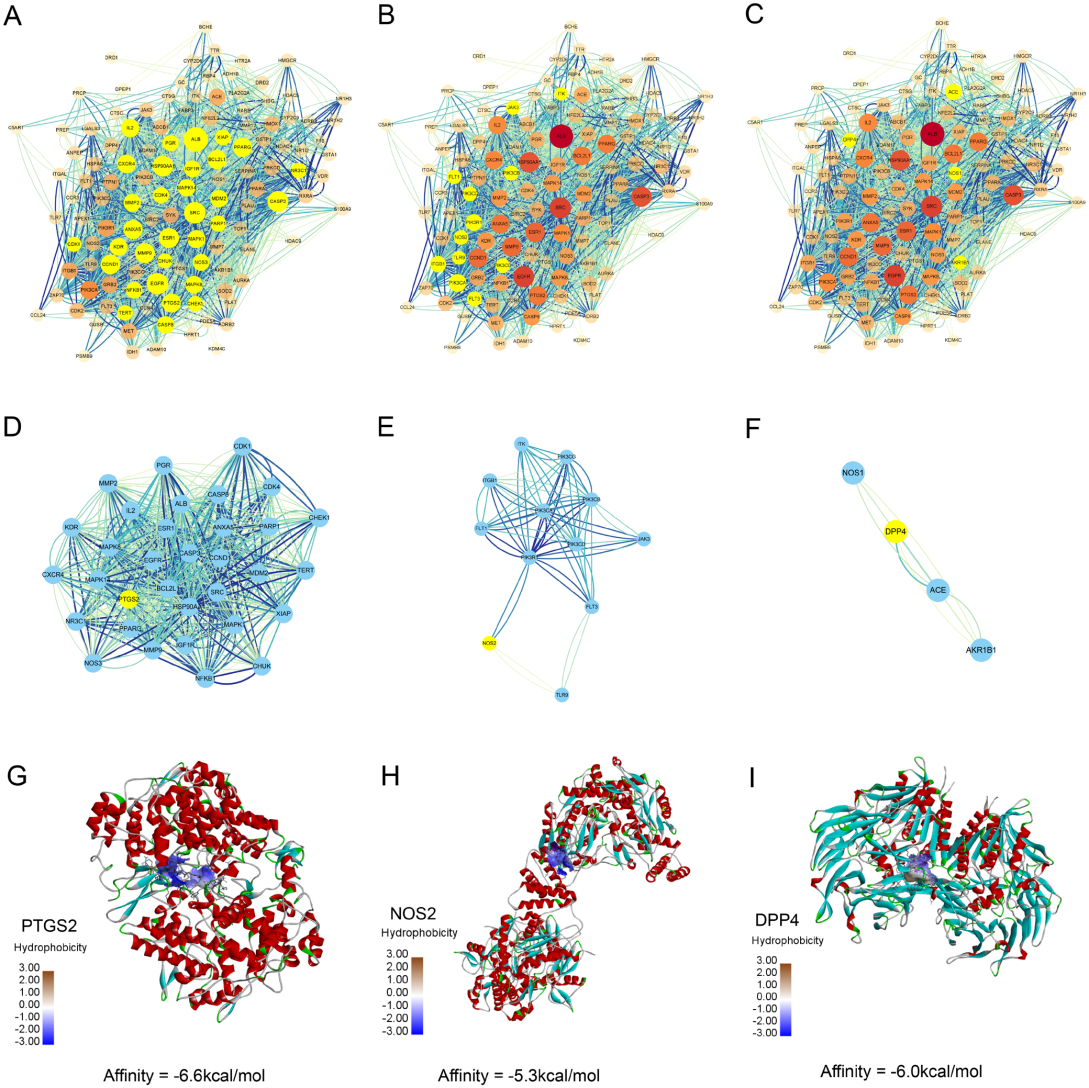
Cluster analysis of the PPI network of HCQ targets related to IgAN and seed gene identification. (A–C) Three main clusters in the original PPI network (The size of the circle represents the degree, and the yellow circles represent genes of corresponding clusters analysed by MCODE). (D–F) The main three clusters analysed by MCODE (The yellow circle represents the seed gene). (G–I) Molecular docking results of affinity between HCQ and PTGS2, NOS2 and DPP4.

To further investigate the detailed mechanism of HCQ against IgAN, a PPI network of HCQ targets related to IgAN was constructed and key targets were screened. Three main clusters were demonstrated in Figure 4A–F, and the seed genes were PTGS2, NOS2, and DPP4, among which the PTGS2 cluster has the most proteins. Furthermore, the results of molecular docking between HCQ and PTGS2, NOS2, and DPP4 revealed that the affinities were − 6.6, −5.3, and − 6.0 kcal/mol respectively (Figure 4G–I), indicating that HCQ and PTGS2 have the highest affinity. These results suggest that the therapeutic effect of HCQ on IgAN may be mainly achieved by targeting PTGS2.

### 3.5 MD Simulations Results of HCQ‐PTGS2 Complex

**FIGURE 5:**
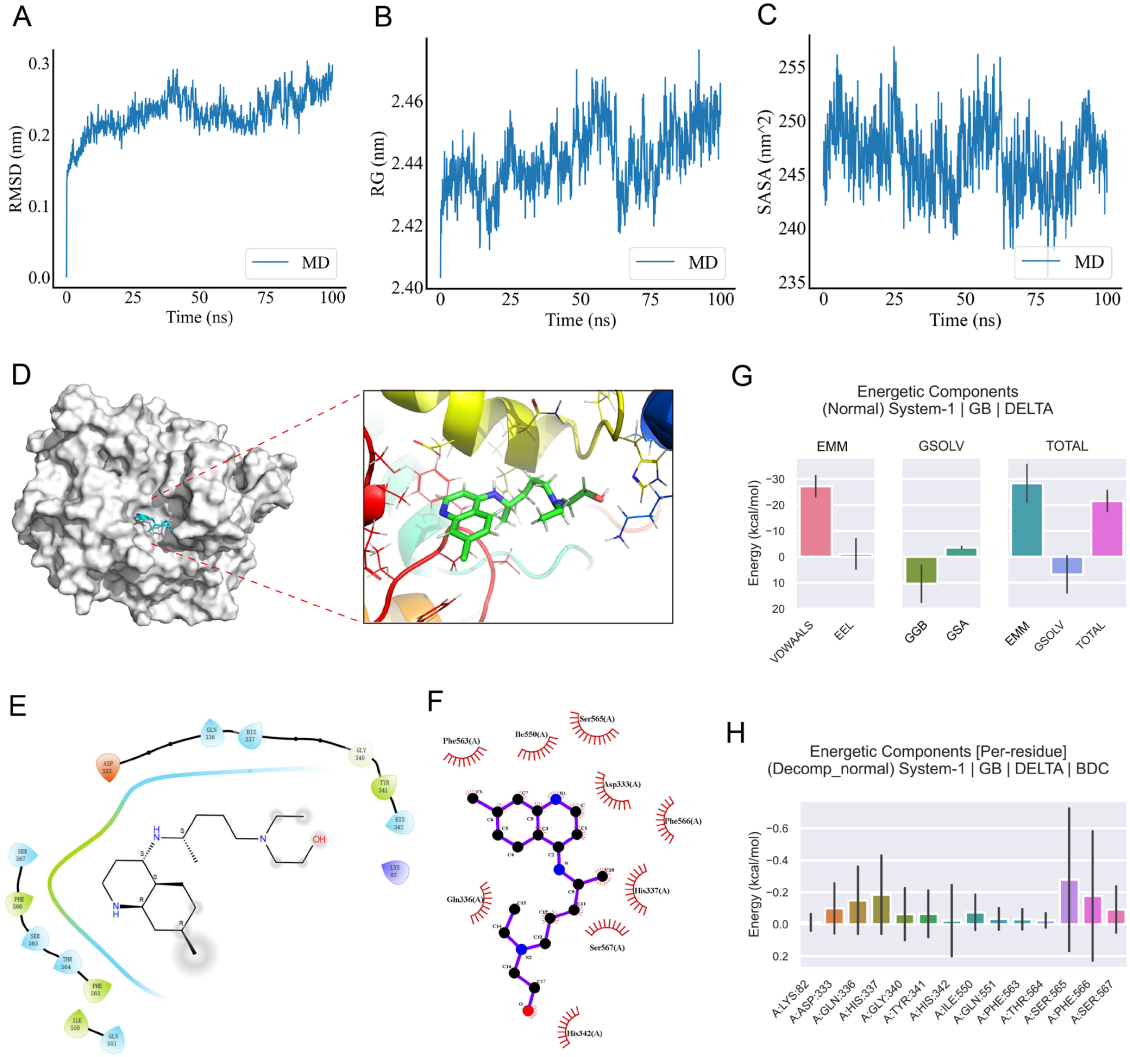
Results of Molecular docking and MD simulations of HCQ‐PTGS2 complex. RMSD (A), RG (B) and SASA (C) profile of the HCQ in complex with PTGS2 during the process of MD simulations. (D) Binding mode of HCQ and PTGS2 during MD simulations (The left showed the global graph, and the right presented the local graph). (E) Interaction diagram between HCQ and surrounding amino acids of PTGS2 protein (Blue, green, red and purple colour represent polar amino acid, hydrophobic amino acid, negative amino acid and positive amino acid respectively; and the arrow form ligand to receptor). (F) The specific view of the 2‐D ligand interaction among HCQ with PTGS2. (G) Binding free energies and energy components calculated by gmx MMPBSA. (H) Decomposition energy of different amino acid residues.

To verify the reliability of HCQ binding to PTGS2, MD simulations were performed, and RMSD, RG, and SASA were calculated. The RMSD of MD simulations could reflect the movement process of the complex, and the result showed that the HCQ‐PTGS2 complex reached about 0.25 nm after 50 ns, with small fluctuations in subsequent simulations (Figure 5A). The RG is an important indicator for evaluating complex complexity. Consistent with the RMSD results, RG first increased and then decreased and fluctuated within a small range in the entire simulation (Figure 5B). Additionally, the SASA of the HCQ‐PTGS2 complex remained stable and was maintained at 240 nm^2 (Figure 5C).

Subsequently, the interaction modes of HCQ and PTGS2 were analysed. The results revealed that HCQ was parked into the active cavity of the PTGS2 protein (Figure 5D). Further analysis of the interaction force demonstrated that a hydrophobic force was formed between HCQ and amino acid residues of the PTGS2 protein, including ASP‐333, GLN‐336, HIS‐337, HIS‐342, ILE‐550, PHE‐563, SER‐565, PHE‐566, and SER‐567 (Figure 5E,F).

Finally, the binding free energy and energy components were computed based on the MD simulations. Generally, a negative value indicates that the molecule has binding affinity with the target protein. As presented (Figure 5G and Table S1), the total binding free energy for the HCQ‐PGS2 complex was −21.57 kcal/mol, suggesting a good binding. For the complex, the van der Waals energy (VDWAALS) played the most important role in contributing to the total binding energy, followed by the non‐polar solvation free energy (GSA), while the solvation free energy (GGB) did not contribute to the complex free energy. Analysis of the decomposition energy of the amino acid residues showed that the main residues contributing to the total binding free energy were SER:565, HIS:337, PHE:566, and GLN:336 (Figure 5H and Table S2).

### 3.6 PTGS2 Overexpressed in IgAN Patients and Was Negatively Related to eGFR

**FIGURE 6:**
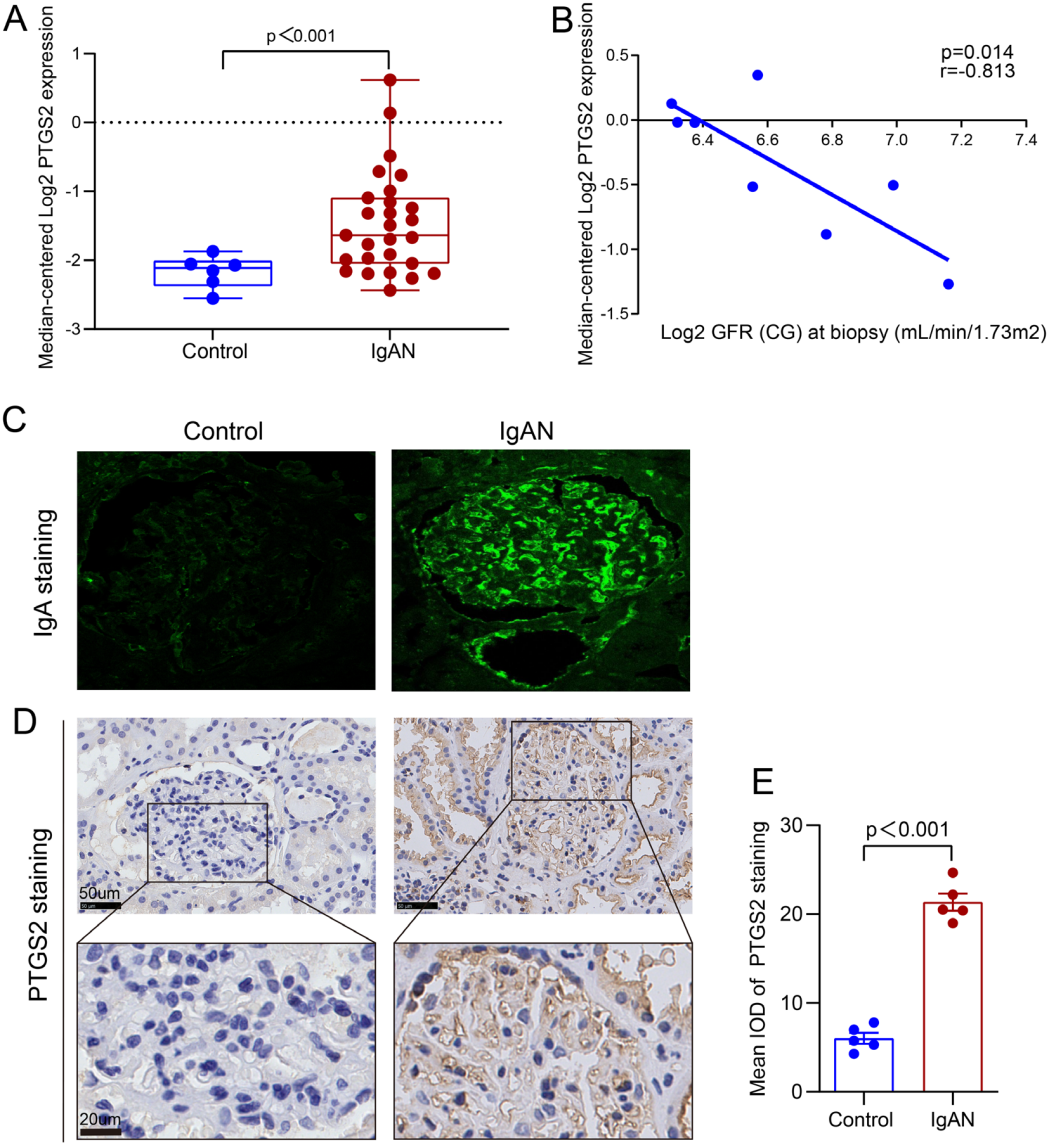
PTGS2 overexpressed in IgAN patients and was negatively related to eGFR. (A) PTGS2 expression (median‐centred log_2_) in kidneys from IgAN patients (*n* = 27) vs. healthy living donors (*n* = 6) (data extracted from Nephroseq ‘Reich IgAN Glom’ datasets). (B) Blood PTGS2 expression level was negatively related to eGFR in IgAN patients (data extracted from Nephroseq ‘Cox IgAN Blood’ datasets, *n* = 8, *p* = 0.014, *r* = −0.813). (C) Representative images of IgA immunofluorescence staining in kidneys from controls and IgAN patients (×400). (D and E) Representative images of PTGS2 immunohistochemical staining in kidneys from controls and IgAN patients and quantitative analysis (*n* = 5) (Up: Scale bar = 50um; Down: Scale bar = 20um).

To explore the role of PTGS2 in the pathogenesis of IgAN, PTGS2 expression in patients with IgAN was evaluated. First, we analysed existing data from the renal transcriptomics database Nephroseq. Compared with the controls, PTGS2 expression levels were evidently increased in the kidneys of IgAN patients (Figure 6A). Moreover, blood PTGS2 expression levels were negatively correlated with eGFR in IgAN patients (Figure 6B). Additionally, five pairs of renal samples from IgAN patients and controls were enrolled in this study, and the general information of the participants is presented in Table S3. Immunofluorescence staining revealed that IgA was significantly deposited in the glomerular mesangial area of IgAN patients compared to that in the control (Figure 6C). Consistent with the Nephroseq database findings, IHC staining revealed elevated PTGS2 expression in the renal tissues of patients with IgAN compared to controls (Figure 6D,E). These findings suggest that PTGS2 may play a pivotal role in the pathogenesis of IgAN.

### 3.7 HCQ Decreased the Expression of PTGS2 and Profibrotic Proteins in HMCs Treated With pIgA1

**FIGURE 7:**
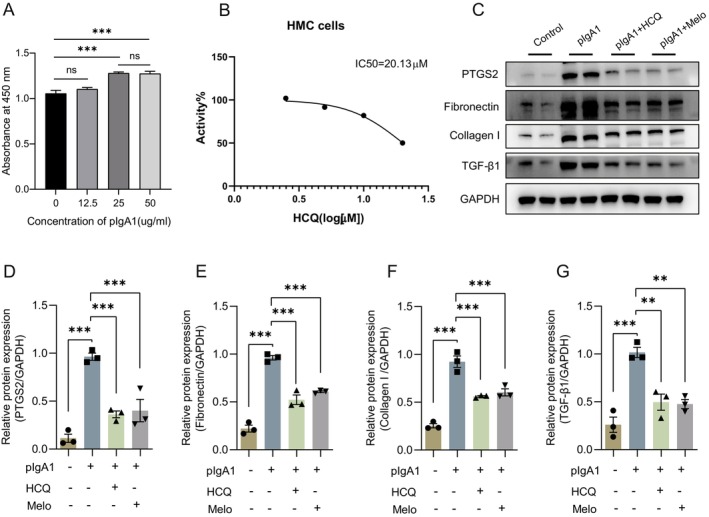
HCQ decreased the expression of PTGS2 and profibrotic proteins in HMCs treated with pIgA1. (A) HMCs were stimulated with different concentrations of pIgA1 to construct IgAN model (*n* = 6). (B) Cell viability was measured by CCK‐8 assay after incubating HMCs at different concentrations of HCQ for 48 h. (C–G) PTGS2, Fibronectin, Collagen I and TGF‐β1 protein levels of cells in different groups analysed by western blotting and quantitative analysis (*n* = 3). Melo, meloxicam; **, *p* < 0.01; ***, *p* < 0.001.

To further verify whether the therapeutic effect of HCQ on IgAN is achieved by binding to PTGS2 and inhibiting its action, in vitro experiments were conducted. First, different concentrations of pIgA1 were used to stimulate HMCs, and 25ug/mL was found to be the most suitable concentration to construct an IgAN model (Figure 7A). Then, the safe dose of HCQ on HMCs was determined using CCK‐8, and results showed that the IC50 value of HCQ was 20.13 μM (Figure 7B). As profibrotic factors secreted by mesangial cells play a significant role in the development of IgAN [33], we then evaluated the effect of HCQ on PTGS2 and profibrotic proteins in HMCs treated by pIgA1. Consistent with the effect of meloxicam, a PTGS2 inhibitor, HCQ could decrease the expression levels of PTGS2 and profibrotic proteins in HMCs treated by pIgA1 (Figure 7C–G). These results suggest that HCQ probably inhibits the activity of PTGS2, thereby alleviating renal fibrosis.

## 4 Discussion

This study sought to clarify the potential therapeutic mechanism of HCQ against IgAN by investigating the intersection of IgAN‐related genes and HCQ targets, followed by virtual simulation experiments, clinical data, and in vitro experiments for verification. Molecular docking and MD simulation analyses indicated that HCQ bound well to the key target, PTGS2. Clinical data and in vitro experiments suggested that HCQ probably exerted therapeutic effects by inhibiting the function of PTGS2, thereby alleviating renal fibrosis.

IgAN is defined by the presence of IgA‐dominant or co‐dominant immune deposits in the mesangial area of the glomeruli [14]. Although the exact pathogenesis of IgAN remains obscure, it is generally considered that the proliferation and phenotypic transformation of mesangial cells and subsequent inflammatory and immune responses play key roles in renal injury [33]. In this study, 1575 IgAN‐related genes were identified. Enrichment analysis revealed that these genes were mainly enriched in inflammation‐nd immune response‐related signalling pathways, which is consistent with previous studies.

HCQ is derived from chloroquine (CQ), an analog of quinine, which was originally extracted from the bark of cinchona in 1820 [34]. Compared to CQ, HCQ has fewer side effects; thus, it is widely used in rheumatic autoimmune disorders and skin diseases [35]. HCQ has been reported to regulate four sets of cellular functions, including inhibition of lysosomal activity and autophagy, NADPH oxidase, cytokine signalling pathways, and calcium signalling pathways, thereby mediating anti‐inflammatory and immune regulatory functions [9, 36]. In line with previous reports, our results suggested that HCQ targets were mainly enriched in the PI3K‐Akt signalling pathway, C‐type lectin receptor signalling pathway, calcium signalling pathway, T cell receptor signalling pathway, TNF signalling pathway, and so on, which are closely related to inflammation and immune response regulation [29, 30, 32].

Several clinical studies have demonstrated that HCQ could effectively reduce proteinuria in IgAN patients and delay the progression of IgAN [5, 6, 7]. Few studies have suggested that HCQ treatment could suppress NF‐κB signalling and NLRP3 inflammasome activation and regulate the differentiation of CD4+ T cell subsets in the kidneys of IgAN rats. However, the exact molecular mechanism of HCQ in IgAN remains unknown [10, 11]. In this study, we systematically analysed the potential targets of HCQ against IgAN through network pharmacology and bioinformatics and found that these targets were mainly enriched in inflammation and immune response‐related signalling pathways, which was consistent with previous reports [9, 10, 11]. Furthermore, via cluster analysis and seed gene identification, we found that PTGS2 is probably the key target of HCQ against IgAN. Additionally, molecular docking and MD simulations showed that HCQ could bind to PTGS2 and influence PTGS2 spatial conformation, which, to some extent, verified the hypothesis that PTGS2 is a potential key target of HCQ against IgAN.

PTGS2, also known as cyclooxygenase 2 (COX‐2), is a key enzyme that converts arachidonic acid into prostaglandins (PGs). PGs mediate diverse cellular biological functions, such as cell differentiation, proliferation, and apoptosis [37, 38, 39], and play critical roles in the pathological processes of inflammation, cancer, renal injury, and cardiovascular diseases [40, 41, 42, 43]. Galactose‐deficient IgA1 (Gd‐IgA1) can activate mesangial cells (MCs), leading to hyperproliferation and secretion of pro‐inflammatory cytokines and profibrotic components such as IL‐1β, IL‐6, TNF, transforming growth factor‐β (TGF‐β), and fibronectin, which in turn stimulate the expression of PTGS2 and prostaglandin synthesis [44, 45]. Meanwhile, PGs crosstalk with pro‐inflammatory cytokines amplifies cytokine actions on various types of inflammatory cells and drives pathogenic conversion of these cells [40]. PGs and cytokines synergistically activate NF‐κB to induce the expression of inflammation‐related genes, while PTGS2 is one of the most important factors that form PG‐mediated positive feedback loops [40]. Our study demonstrated that PTGS2 was overexpressed in the renal tissues of patients with IgAN, and blood PTGS2 levels were negatively correlated with eGFR. Accordingly, inhibition of PTGS2 could reduce indoxyl sulfate and inflammatory conditions induced mesangial cell proliferation [46]. Meanwhile, a previous study revealed that meloxicam, a PTGS2 inhibitor, could alleviate mouse renal fibrosis induced by UUO through decreasing the expression of HSP47 and phosphorylation of extracellular regulated kinase (ERK) and c‐jun‐N‐terminal kinase (JNK), which play significant roles in the process of inflammation [47]. Additionally, our in vitro experiments demonstrated that, consistent with the effect of meloxicam, HCQ could decrease the expression of PTGS2 and profibrotic proteins in HMCs treated by pIgA1. Together, these results indicated that HCQ probably inhibits the activity of PTGS2 by binding to it, thereby alleviating renal fibrosis and delaying the progression of IgAN.

This study has some limitations. First, because of the generality of network pharmacology research, some target genes of HCQ may not have been included in public databases. Second, although the binding of HCQ and PTGS2 was verified by molecular docking and MD simulations, it would be more accurate if direct evidence were added, such as surface plasmon resonance analysis. Third, it is better to supplement in vivo studies to further verify the potential mechanism of HCQ against IgAN. These limitations will be the focus of our future studies.

## 5 Conclusion

In summary, this study systematically revealed the potential mechanism by which HCQ can treat IgAN, probably involving multiple targets and signalling pathways that are mainly related to inflammation and immune response regulation. Among the multiple targets, further studies found that PTGS2 was probably the key target of HCQ against IgAN, and HCQ potentially exerted pharmacological effects by binding to PTGS2 to inhibit its activity. These findings provide a theoretical foundation for the clinical application of HCQ in IgAN and offer insights for future research into its therapeutic mechanisms.

## Author Contributions

**Yuyuan Liu:** conceptualization (equal), data curation (equal), formal analysis (equal), funding acquisition (equal), investigation (equal), methodology (equal), visualization (equal), writing – original draft (equal). **Jinfang Hu:** formal analysis (equal), investigation (equal), methodology (equal), validation (equal), writing – original draft (equal). **Jialing Wang:** investigation (equal), methodology (equal). **Yanzhe Wang:** formal analysis (equal), project administration (equal), supervision (equal), visualization (equal), writing – review and editing (equal). **Gang Wu:** conceptualization (equal), funding acquisition (equal), project administration (equal), supervision (equal), writing – review and editing (equal).

## Conflicts of Interest

The authors declare no conflicts of interest.

## Supporting information


**Table S1.** Binding free energies and energy components calculated by gmx_MMPBSA.

**Table S2.** Analysis of decomposition energy of amino acid residues.

**Table S3.** Clinical data of human participants.

## Data Availability

The data that support the findings of this study are available from the corresponding author upon reasonable request.
