# Supplementary material for: Exploring the Molecular Mechanism of Hydroxychloroquine Against IgAN Through Network Pharmacology, MD Simulations and Experimental Assessment
Source: J Cell Mol Med. 2025 May 26;29(10):e70615. doi: 10.1111/jcmm.70615 (PMC12105495; doi:10.1111/jcmm.70615)
Supplement: Supplementary file 1 — Table S1. Binding free energies and energy components calculated by gmx_MMPBSA. [file JCMM-29-e70615-s003.docx]

**Table S1. Binding free energies and energy components calculated by gmx_MMPBSA.**

| System name | HC/PTGS2(kcal/mol) |
| --- | --- |
| **ΔE_vdw_** | -27.25 |
| **ΔE_elec_** | -1.12 |
| **ΔG_GB_** | 10.38 |
| **ΔG_SA_** | -3.58 |
| **ΔE_MM_** | -28.37 |
| **ΔG_SOLV_** | 6.80 |
| **ΔG_bind_(Total)** | -21.57 |

ΔE_VDW_: Van der Waals energy; ΔE_elec_: electrostatic energy; ΔG_GB_: solvation free energy; ΔG_SA_: non-polar solvation free energy; ΔE_MM_: equal the sum of ΔE_VDW_ and ΔE_elec_; ΔG_SOLV_: equal the sum of ΔG_GB_ and ΔG_SA_; ΔG_bind_: binding free energy.
