# Supplementary material for: Exploring the Molecular Mechanism of Hydroxychloroquine Against IgAN Through Network Pharmacology, MD Simulations and Experimental Assessment
Source: J Cell Mol Med. 2025 May 26;29(10):e70615. doi: 10.1111/jcmm.70615 (PMC12105495; doi:10.1111/jcmm.70615)
Supplement: Supplementary file 2 — Table S2. Analysis of decomposition energy of amino acid residues. [file JCMM-29-e70615-s001.docx]

**Table S2. Analysis of decomposition energy of amino acid residues**

| Frames | R:A:LYS:82 | R:A:ASP:333 | R:A:GLN:336 | R:A:HIS:337 | R:A:GLY:340 | R:A:TYR:341 | R:A:HIS:342 | R:A:ILE:550 | R:A:GLN:551 | R:A:PHE:563 | R:A:THR:564 | R:A:SER:565 | R:A:PHE:566 | R:A:SER:567 |
| --- | --- | --- | --- | --- | --- | --- | --- | --- | --- | --- | --- | --- | --- | --- |
| 1 | -0.03 | -0.49 | -0.2 | -0.46 | -0.57 | -0.09 | -0.19 | -0.23 | -0.04 | -0.22 | -0.01 | -0.97 | 0.06 | -0.3 |
| 101 | -0.17 | -0.23 | -0.24 | -0.47 | -0.06 | -0.15 | -0.05 | -0.28 | -0.2 | -0.05 | -0.15 | -1.38 | -1.4 | -0.45 |
| 201 | 0 | -0.15 | -0.71 | -0.66 | -0.11 | 0.27 | -0.29 | -0.04 | 0.02 | -0.01 | -0.03 | -0.38 | -0.31 | -0.18 |
| 301 | 0 | -0.25 | -0.33 | -0.41 | 0.01 | -0.38 | 0.64 | -0.01 | 0.01 | 0 | 0 | -0.16 | -0.28 | -0.05 |
| 401 | 0.03 | 0.04 | -0.13 | -0.05 | -0.01 | -0.17 | -0.01 | -0.26 | -0.14 | -0.05 | -0.08 | -0.18 | -0.03 | -0.03 |
| 501 | 0.01 | 0 | 0 | 0 | 0.01 | -0.01 | -0.05 | 0 | 0 | 0 | 0 | 0 | 0 | 0 |
| 601 | 0.01 | 0 | -0.01 | 0 | 0 | -0.03 | -0.03 | 0 | 0 | 0 | 0 | 0 | 0 | 0 |
| 701 | 0 | 0 | -0.01 | 0 | 0 | -0.03 | -0.05 | 0 | 0 | 0 | 0 | 0 | 0 | 0 |
| 801 | 0.01 | 0 | 0 | 0 | 0.01 | -0.04 | -0.08 | 0 | 0 | 0 | 0 | 0 | 0 | 0 |
| 901 | 0.01 | 0 | 0 | 0 | 0 | -0.05 | -0.06 | 0 | 0 | 0 | 0 | 0 | 0 | 0 |
| 1001 | 0 | 0 | -0.01 | 0 | 0.01 | -0.04 | -0.07 | 0 | 0 | 0 | 0 | 0 | 0 | 0 |
|  | -0.13 | -1.08 | -1.64 | -2.05 | -0.71 | -0.72 | -0.24 | -0.82 | -0.35 | -0.33 | -0.27 | -3.07 | -1.96 | -1.01 |
