# Supplementary material for: Exploring the Molecular Mechanism of Hydroxychloroquine Against IgAN Through Network Pharmacology, MD Simulations and Experimental Assessment
Source: J Cell Mol Med. 2025 May 26;29(10):e70615. doi: 10.1111/jcmm.70615 (PMC12105495; doi:10.1111/jcmm.70615)
Supplement: Supplementary file 3 — Table S3. Clinical data of human participants. [file JCMM-29-e70615-s002.docx]

**Table S3. Clinical data of human participants**

| Group | Gender | Age  (years) | Systolic pressure  (mmHg) | Diastolic pressure  (mmHg) | Serum creatinine  (μmol/L) | Blood urea nitrogen  (mmol/L) | Serum uric acid (μmol/L) | eGRF (CKD-EPI, ml/min) | Urinary albumin/creatinine ratio(mg/g) | 24h urinary total protein(g/24h) | Serum IgA(g/L) | Serum IgG4(g/L) |
| --- | --- | --- | --- | --- | --- | --- | --- | --- | --- | --- | --- | --- |
| Control-1 | Femal | 55 | 122 | 71 | 65.2 | 5.42 | 299.6 | 91.57 | 24.16 | - | - | - |
| Control-2 | Male | 65 | 125 | 80 | 82.7 | 7.65 | 388.4 | 85.23 | 18.23 | - | - | - |
| Control-3 | Femal | 54 | 110 | 65 | 72 | 4.84 | 232.6 | 81.72 | 28.48 | - | - | - |
| Control-4 | Male | 46 | 128 | 70 | 68.3 | 6.58 | 361.3 | 108.67 | 13.87 | - | - | - |
| Control-5 | Male | 53 | 132 | 64 | 85.6 | 6.26 | 405.7 | 88.94 | 25.64 | - | - | - |
| IgAN-1 | Femal | 31 | 121 | 68 | 93 | 7.04 | 577.5 | 70.55 | 154.97 | 0.25 | 4.53 | Normal |
| IgAN-2 | Femal | 46 | 135 | 90 | 83 | 5.46 | 290.8 | 72.85 | 187.85 | 0.8 | 3.54 | Normal |
| IgAN-3 | Femal | 59 | 118 | 76 | 78.1 | 10.11 | 292.9 | 71.57 | 95.83 | 0.26 | 2.53 | Normal |
| IgAN-4 | Male | 33 | 116 | 98 | 82 | 5.44 | 322.7 | 107.82 | 140.03 | 0.26 | 1.96 | Normal |
| IgAN-5 | Male | 37 | 117 | 74 | 100 | 5.57 | 319.3 | 82.47 | 502.99 | 0.65 | 2.91 | Normal |

Table continued on next page.

| Group | PLA2R | ANCA | ANA* | Ati-dsDNA  antibody | Ati-Sm  antibody | Ati-SSA   antibody | Ati-SSB  antibody | Ati-GBM  antibody | Viral hepatitis* | HIV | Syphilis  trust | Pathologic diagnosis |
| --- | --- | --- | --- | --- | --- | --- | --- | --- | --- | --- | --- | --- |
| Control-1 | - | - | - | - | - | - | - | - | Negative | Negative | Negative | Normal  (without cancer cell infiltration) |
| Control-2 | - | - | - | - | - | - | - | - | Negative | Negative | Negative | Normal  (without cancer cell infiltration) |
| Control-3 | - | - | - | - | - | - | - | - | Negative | Negative | Negative | Normal  (without cancer cell infiltration) |
| Control-4 | - | - | - | - | - | - | - | - | Negative | Negative | Negative | Normal  (without cancer cell infiltration) |
| Control-5 | - | - | - | - | - | - | - | - | Negative | Negative | Negative | Normal  (without cancer cell infiltration) |
| IgAN-1 | Negative | Negative | Negative | Negative | Negative | Negative | Negative | Negative | Negative | Negative | Negative | IgAN |
| IgAN-2 | Negative | Negative | Negative | Negative | Negative | Negative | Negative | Negative | Negative | Negative | Negative | IgAN |
| IgAN-3 | Negative | Negative | Negative | Negative | Negative | Negative | Negative | Negative | Negative | Negative | Negative | IgAN |
| IgAN-4 | Negative | Negative | Negative | Negative | Negative | Negative | Negative | Negative | Negative | Negative | Negative | IgAN |
| IgAN-5 | Negative | Negative | Negative | Negative | Negative | Negative | Negative | Negative | Negative | Negative | Negative | IgAN |

*****ANA, Anti-nuclear antibody; Viral hepatitis, (including HBV and HCV).
